# Intelligent Mining of Urban Ventilation Corridors Based on High-Precision Oblique Photographic Images

**DOI:** 10.3390/s21227537

**Published:** 2021-11-12

**Authors:** Chaoxiang Chen, Shiping Ye, Zhican Bai, Juan Wang, Zongbiao Zhang, Sergey Ablameyko

**Affiliations:** 1 School of Information Science and Technology, Zhejiang Shuren University, Hangzhou 310000, China; ccx@zjsru.edu.cn (C.C.); 600352@zjsru.edu.cn (Z.B.); juanw@zjsru.edu.cn (J.W.); zzb@zjsru.edu.cn (Z.Z.); 2 International Science and Technology Cooperation Base of Zhejiang Province: Remote Sensing Image Processing and Application, Hangzhou 310000, China; ablameyko@bsu.by; 3 Faculty of Applied Mathematics and Computer Science, Belarusian State University, 220004 Minsk, Belarus; 4 United Institute of Informatics Problems of National Academy of Sciences, 220004 Minsk, Belarus

**Keywords:** urban ventilation corridors, intelligent mining, template matching, high-precision oblique photographic image

## Abstract

With the advancement of urbanization and the impact of industrial pollution, the issue of urban ventilation has attracted increasing attention. Research on urban ventilation corridors is a hotspot in the field of urban planning. Traditional studies on ventilation corridors mostly focus on qualitative or simulated research on urban climate issues such as the intensified urban heat island effect, serious environmental pollution, and insufficient climate adaptability. Based on the high-precision urban remote sensing image data obtained by aeromagnetic oblique photography, this paper calculates the frontal area density of the city with reference to the urban wind statistics. Based on the existing urban patterns, template matching technology was used to automatically excavate urban ventilation corridors, which provides scientific and reasonable algorithmic support for the rapid construction of potential urban ventilation corridor paths. It also provides technical methods and decision basis for low-carbon urban planning, ecological planning and microclimate optimization design. This method was proved to be effective through experiments in Deqing city, Zhejiang Province, China.

## 1. Introduction

As society develops, the global urbanization process is rapidly increasing. It is predicted that China’s urbanization rate will reach more than 70% in 2050 [1]. Rapid urbanization has strongly promoted the sustainable development of an economical society and the overall improvement in the standards of living for urban and rural residents. However, high-speed urbanization has also caused urban environmental problems such as the deterioration of the microclimate environment and the serious urban heat island effect. Relevant studies have shown that good urban ventilation can promote urban air circulation and reduce air pollution [2,3,4]. Improving the urban ventilation environment and building urban ventilated corridor are effective measures to improve urban air circulation capacity and alleviate urban environmental problems [5,6,7].

Both domestic and international leading research institutes conduct ventilated corridor research using three main methods: field measurements, physical simulation tests, and computer numerical simulation analyses [8,9]. Comparatively, based on an urban spatial data model, the method of using computer analysis technology to assist in the planning and design of ventilation corridors has a low cost and short cycle. In addition, it can be evaluated by parameter research via setting conditions, which will play an important role in supporting decisions to improve the overall ventilation efficiency of a city. The development of the Internet of Things (IoT) in recent years [10,11], has provided strong support for urban ventilation corridor studies in terms of data.

In recent years, many scholars have conducted research in this field. Some studies used CFD (computational fluid dynamics) software simulations, remote sensing image inversion, and GIS analysis to explore the planning and design of central urban air ducts [12,13,14]. However, these studies lack systematization [15]. At the same time, from the perspective of planning, the literature also lacks systematic thinking to solve urban environmental and ecological problems. Yuan proposed a WRF model and constructed two-level air ducts at certain municipal scales [16]. Zhang extracted high-resolution urban geographic information data and used RS and GIS integration to collect surface information and spatial information on the downtown area of Jinzhong, obtaining an urban roughness pattern map of the downtown area [17]. Generally speaking, however, these studies lack in-depth discussion of the detailed urban form. Moreover, knowledge and redundancy on digging urban ventilation corridors are insufficient.

Taking Deqing County, Huzhou city, Zhejiang Province, as an example, this paper used a Quest Kodiak 100 aircraft equipped with a five-view high-definition tilt aerial camera to obtain tilted image data. Through data processing of the measurements of control point plane coordinates and altimetric data, multi-view tilted images of DSM and DEM were extracted. To be specific, according to statistics from the meteorological department, we analyzed the dominant wind direction of the target city; then, the DSM–DEM map was processed to obtain data such as urban buildings. In addition, through the dominant wind direction projection, we calculated the frontal area density (FAD) map and then further considered information such as the height of zero-plane displacement and the influence of building height on wind power changes. Next, we performed image processing, such as binarization, expansion, corrosion, and template matching, on the FAD map and DSM–DEM map to calculate the urban ventilation corridor data. According to the empirical value of the urban ventilation corridor, the ventilation corridors of the target city were extracted. The experimental results show the effectiveness of this research method.

## 2. City Correlation Analysis and Frontal Area Calculation

### 2.1. Oblique Photography and Urban Digital Data Acquisition

**Figure 1:**
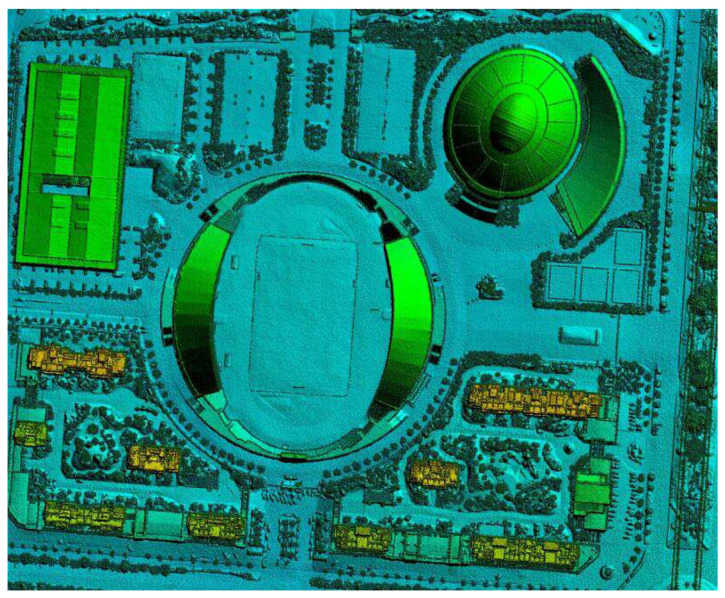
A digital elevation model diagram.

A digital terrain model (DTM) is a database that represents the spatial distribution of ground features. Generally, a series of ground point coordinates (x, y, and z) and surface attributes (target categories, features, etc.) are used to form a data array, so that a digital terrestrial model can be composed. If a terrain feature point only refers to the elevation of the ground point, this digital terrain description can be called a digital elevation model (DEM). A digital surface model (DSM) can be a simulation of the surface’s ground objects including vegetation and houses. A DSM was processed to remove information, such as houses and vegetation, to form a DEM as shown in Figure 1.

Our cooperative partners used the Quest Kodiak 100 aircraft to obtain the tilted image data with a five-view high-definition tilt aerial camera in the early stage. After digital image processing, the city data were geometrically corrected, and a digital ground model (i.e., DSM) was generated. This paper used the difference between the DSM and DEM to extract the height of the surface roughness of buildings, rivers, lakes, and vegetation [18].

### 2.2. Wind Rose Chart and Statistics of the City’s Dominant Directions

**Figure 2:**
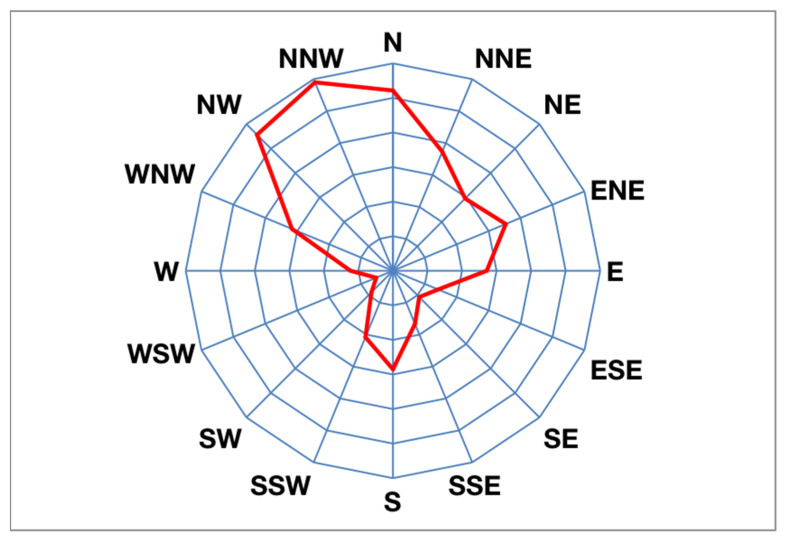
Winter wind rose illustration of Deqing City.

**Figure 3:**
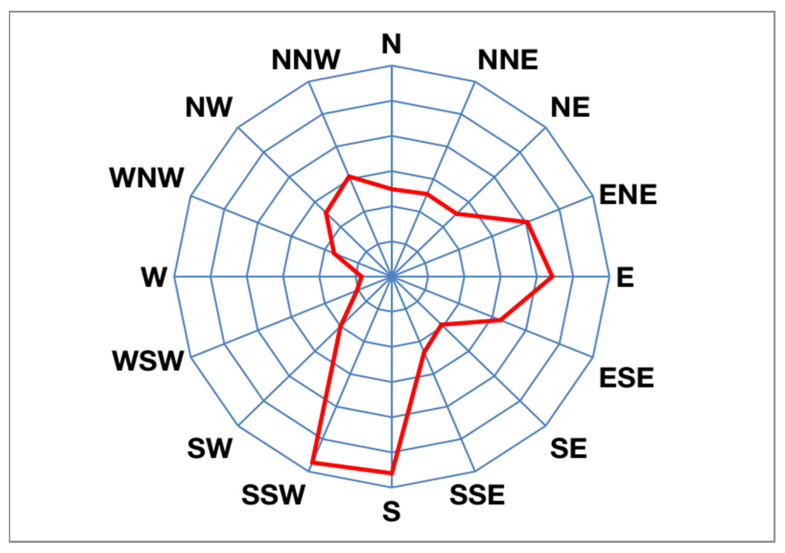
Summer wind rose illustration of Deqing City.

As a professional statistical chart of wind, the wind rose is used to quantitatively analyze the characteristics of wind direction and wind speed over a certain period of time [19]. This study obtained daily average wind speed and wind direction data for the city at a height of 10 m over the past 10 years from the Deqing City Meteorological Department, and it determined the average statistics of the wind frequency in 16 azimuths as shown in Figure 2 and Figure 3, respectively.

In Figure 2, the dominant wind direction in Deqing City during winter is northwest. The frequencies of the NNW, NW, and N wind directions were 11.8%, 11.13%, and 10.43%, respectively, and the sum of the frequencies was 33.36%. In Figure 3, there are two main wind directions: the southerly wind and easterly wind in Deqing City during summer. The frequencies of the SSW and S wind directions were 11.45% and 11.19%, respectively, and the sum of the frequencies was 22.64%. The frequencies of the E, ENE, and ESE wind directions were 8.85%, 8.09%, and 6.48%, respectively, and the sum of the frequencies was 23.42%.

### 2.3. City Ventilation and Frontal Area Density Calculation

Buildings are the most important factor for urban ventilation, and their ventilation evaluation mainly adopts the comprehensive frontal area density method [20]. City frontal area density (FAD) represents a certain height increase, where the ratio is the frontal area of a building under a certain wind condition to the area where the building is located. The equation for calculating the frontal area density is as follows:

(1) $$\;\;\lambda_{f\left(z,\theta \right)}=\frac{A{\left(\theta \right)}_{proj\left(z\right)}}{A_{T}}$$

In Equation (1), A(θ)*_proj(z)_* is the frontal area of the building perpendicular to a certain wind direction; A*_T_* is the area where the building is located; θ is a certain direction selected; z is the height increase (that is, the calculation range to calculate the projected area in the height direction). It can be seen that the frontal area density focuses on the description of the architectural form at the selected specific height increase.

The frontal density *λ_f_* is a parameter related to the wind direction. Different wind directions have a different frontal surface density *λ_f_*. The frontal surface density can reflect the obstructive effect of the urban form on the wind in a specific direction, which reflects the ventilation capacity of the wind in a specific direction in the urban area [21].

**Figure 4:**
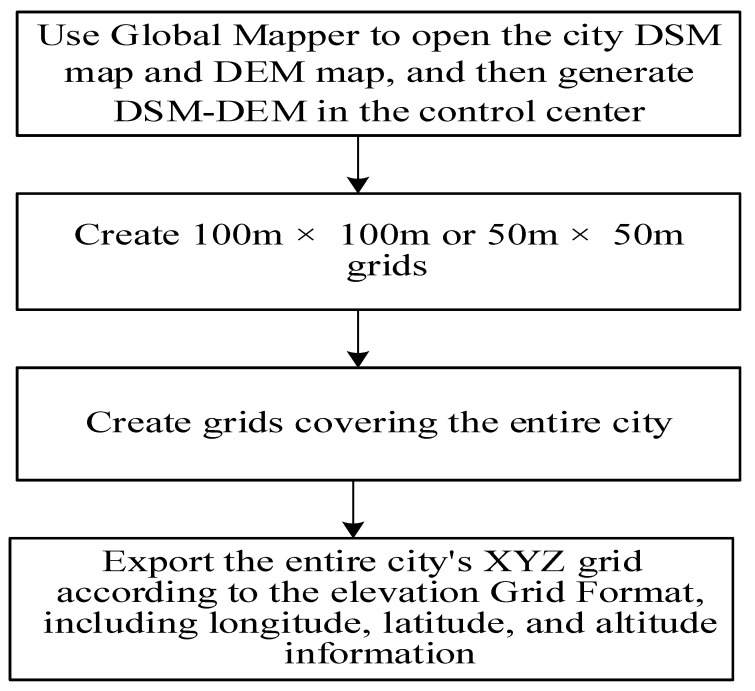
A process using DSM–DEM to extract urban roughness.

In order to calculate the wind density of buildings that dominate the wind direction in winter and summer in the target city, the height of the buildings in the urban area should be classified to count, and then the height increase is determined. The frontal area density focuses on the description of the architectural form at the selected specific height increment. DSM–DEM can be used to extract the elevation of urban roughness as shown in Figure 4.

**Figure 5:**
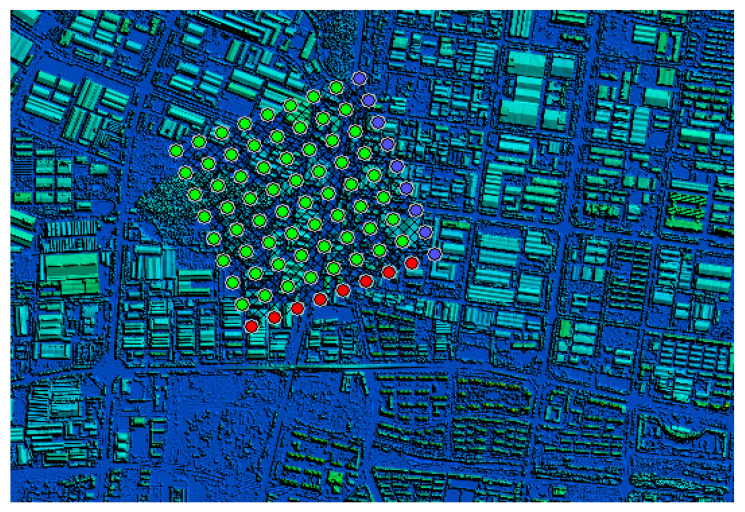
An example of 8 × 8 grids on the city’s DSM–DEM (each grid is 100 × 100 m).

**Figure 6:**
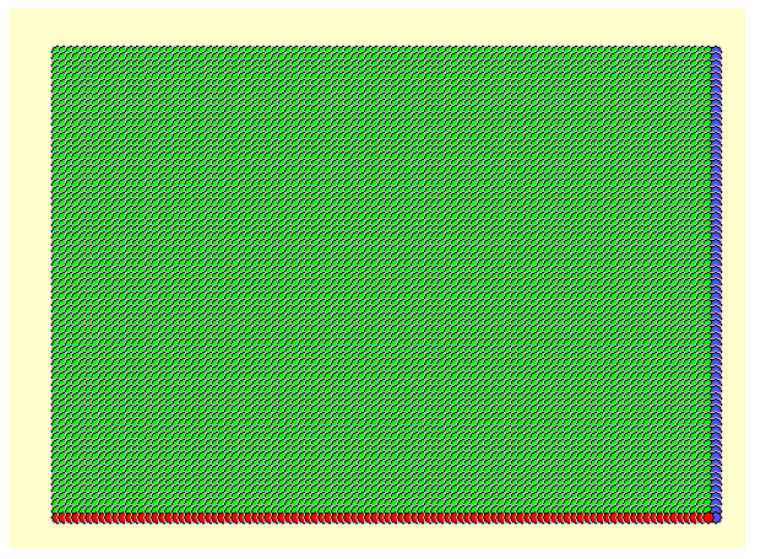
The full coverage map of the grids on the city’s DSM–DEM.

We can use the previous model data (including the high-resolution spatial distribution and height data information of all buildings) to generate 100 × 100 m grid data (as shown in Figure 5). According to the dominant wind direction during winter and summer, through coordinate transformation we can generate a raster image of the city (as shown in Figure 6). Then, we can select the high-frequency wind direction angle of the wind rose map in the winter (similar to summer) of the city and calculate the vertical area of the wind direction for the entire city to increase the height image projection calculation. Next, we can calculate the side area for each grid building, that is, the area of the frontal side, and divide it by the grid area (land parcel), finally automatically calculating the frontal area density of each grid. Because the map faces north, the raster image needs to be rotated (360—the dominant wind angle) for each raster before the FAD calculation, and then rotated back to the north after the calculation is completed. The size of the FAD map is reduced as 1/grid length for both rows and columns compared to the elevation map.

**Figure 7:**
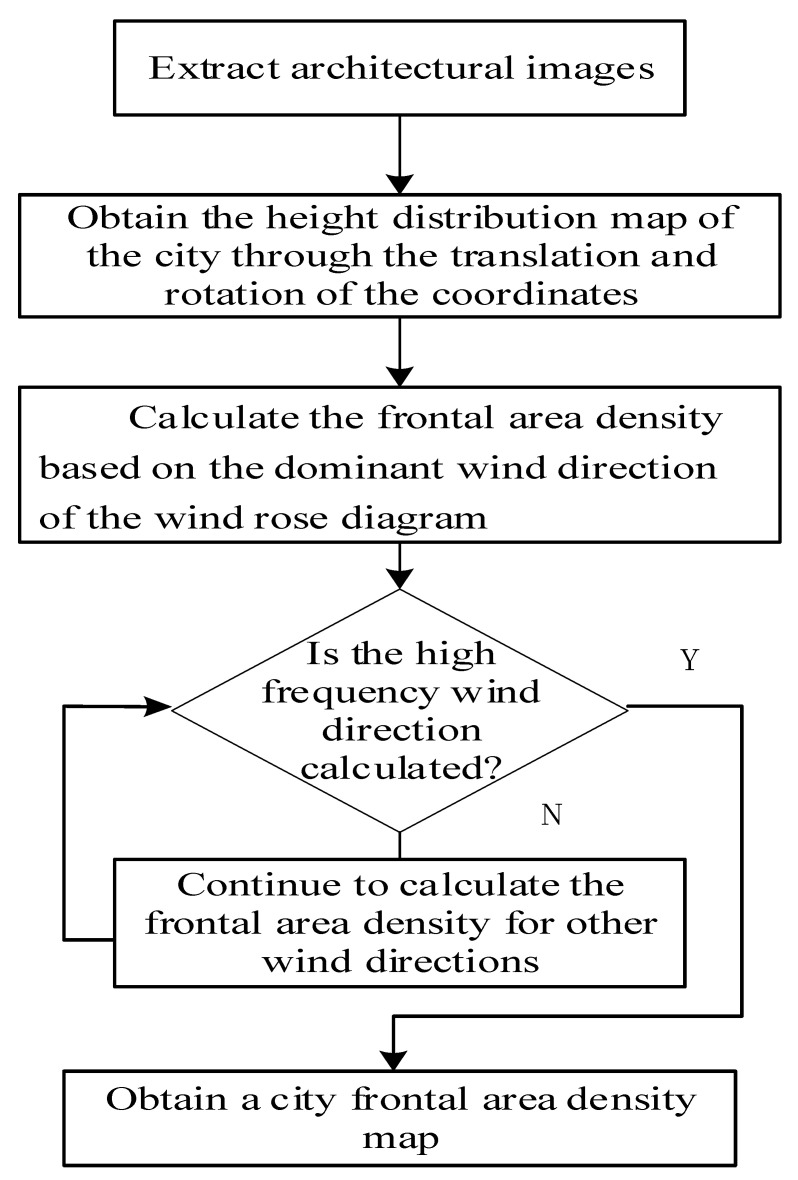
The calculation process of the frontal area density of urban buildings.

The calculation process of the building’s frontal density area is shown in Figure 7.

**Figure 8:**
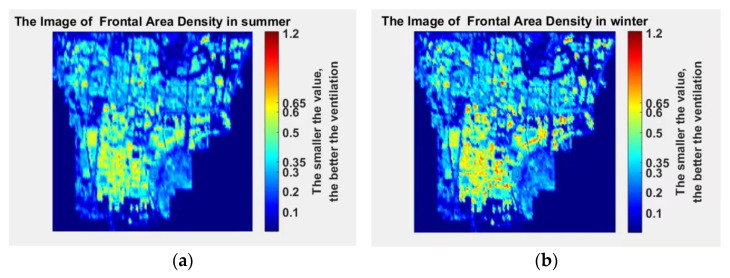
Frontal area density map in the summer and winter: (**a**) summer frontal area density map; (**b**) winter frontal area density map.

The calculated frontal area density in summer and winter are shown in Figure 8a,b, respectively.

## 3. Definition of Urban Ventilation Corridor

Taking the frontal area density as the main calculation factor, the length of the urban ventilation corridor refers to the continuous length of the value of the frontal area density interval [22]. This paper selected the following three conditions as the empirical value for urban ventilation corridors:

(1) VC length > 500 m (preferably 1000 m and above);
(2) VC width ≮ 30 m (building interval, preferably 50 m and above). Among them, the suitable width of an urban air duct was >100 m and a block-scale ventilation corridor VC width ≮ 30 m and a VC width ≯ 100 m;
(3) θ < 45° (where θ is the dominant wind direction of the VC direction), when the urban air duct was parallel to the dominant wind direction, the ventilation efficiency of the air duct was the best, and θ < 30° was more conducive to improvements in the overall wind environment.

**Figure 9:**
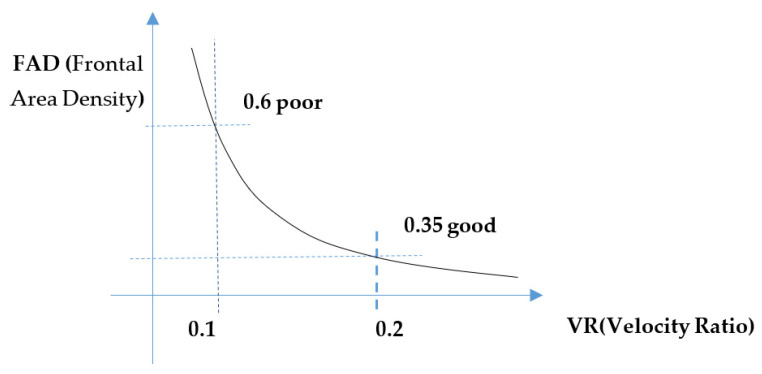
The relationship between frontal area density and velocity ratio [23].

As shown in Figure 9, the smaller the FAD value, the length of VC > 500 m, width of VC > 30 m, and θ < 45, the higher the wind speed and the better the ventilation efficiency. This paper combined the actual target city to divide the urban ventilation corridor grades to CCI, CCII, and CCIII.

The quantitative parameters and qualitative descriptions are as follows:

FAD ≤ 0.35 means natural wind enters smoothly;
0.35 < FAD ≤ 0.45 means that the natural wind does not enter smoothly;
0.45 < FAD ≤ 0.6 means that the natural wind is obstructed;
FAD > 0.6 means that there is a big obstacle for natural wind to enter.

## 4. Intelligent Mining of Urban Ventilation Corridors Based on Templates

Template is a common method in image objective mining, but it has not been used in urban ventilation corridors.

### 4.1. Template Calculation

The template calculation is a type of convolution calculation, which can be regarded as a weighted summation process. Each pixel in the image area used is multiplied by each element of the convolution kernel (weight matrix), and the sum of all products is the new value of the center pixel of the area.

**Figure 10:**
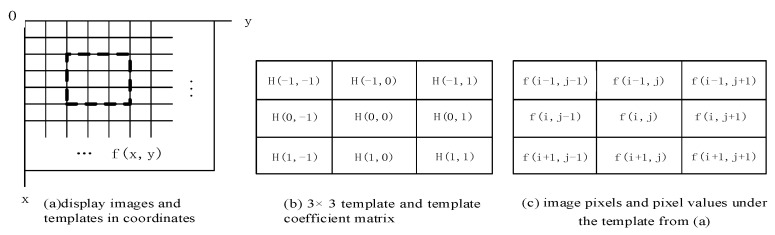
Schematic diagram of the template matching mechanism.

The implementation of template calculation is shown in Figure 10. The dashed black box part of 3 × 3 (i.e., M = 3) in Figure 10a is shown in Figure 10b, H (s,t) (s,t ϵ (−1,+1)) is the value of the 3 × 3 template coefficient.

The method by which the template moves point by point in the image is: the template moves to the right until the last column of the template overlaps the rightmost column of the image; the template moves down until the bottom row of the template overlaps the bottom row of the image. It is defined as:

(2) $$g(i,j)=\sum_{s=-k}^{k}\sum_{t=-l}^{l}f(i+s,j+t)H(s,t)$$

The values of *k* and *l* depend on the size of the selected neighborhood, and at this time *k* = 1. Generally, the values of *k* and *l* are odd numbers, and both *k* and *l* are 1 in Figure 10.

In order to save the loss of the number of rows and columns which is caused by the calculation of Equation (2), we used the method as follows: set the size of the image to be N × N. Next, expand the rightmost side of the original image by M − 1 rows, expand the bottom most side by M − 1 columns, and then respond accordingly The template operation processing can directly obtain the N × N operation result.

Considering the image *f*(*i*,*j*) of degree N × N, there is {*i*,*j* ϵ[0,N − 1]}. For the M × M template {*H*(*s*,*t*)}, there is {*s*,*t* ϵ [0,M − 1]}. Therefore, in the calculation, the value ranges of *s* and *t* can be (0, M − 1), respectively, so that Equation (2) is modified to Equation (3).

(3) $$g(i,j)=\sum_{s=0}^{M}\sum_{t=0}^{M}f(i+s,j+t)H(s,t)\;\{i,j=0,1,2,3,...,N-M\}$$

### 4.2. Template Design

**Figure 11:**
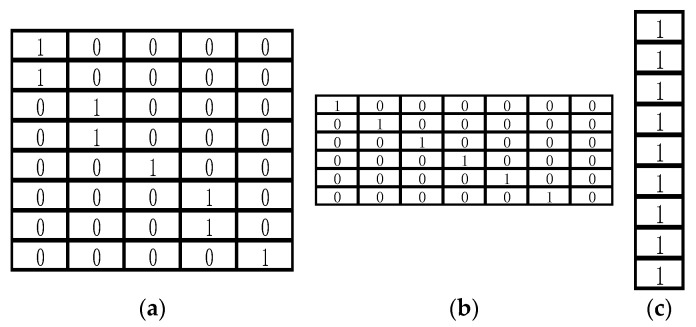
The template design of three dominant wind directions of a winter corridor with the length = 9: (**a**) −67.5 degree template; (**b**) −45 degree template; (**c**) −90 degree template.

Taking winter ventilation corridor mining as an example, according to the wind rose diagram of the target city over the past 10 years, the frequencies of the northwest wind (NNW), northwest wind (NW), and north wind (N) in winter are 11.8%, 11.13%, and 10.43%, respectively. The sum is 33.36%, which satisfies the condition that >30% has a dominant wind direction. Therefore, the dominant wind directions of the city in winter are northwest wind (−67.5 degrees), northwest wind (−45 degrees), and north wind (−90 degrees). For the dominant wind direction, the template was designed as shown in Figure 11 (taking a length of nine as an example).

### 4.3. Automatic Mining Process and Algorithm of the Urban Ventilation Corridor

**Figure 12:**
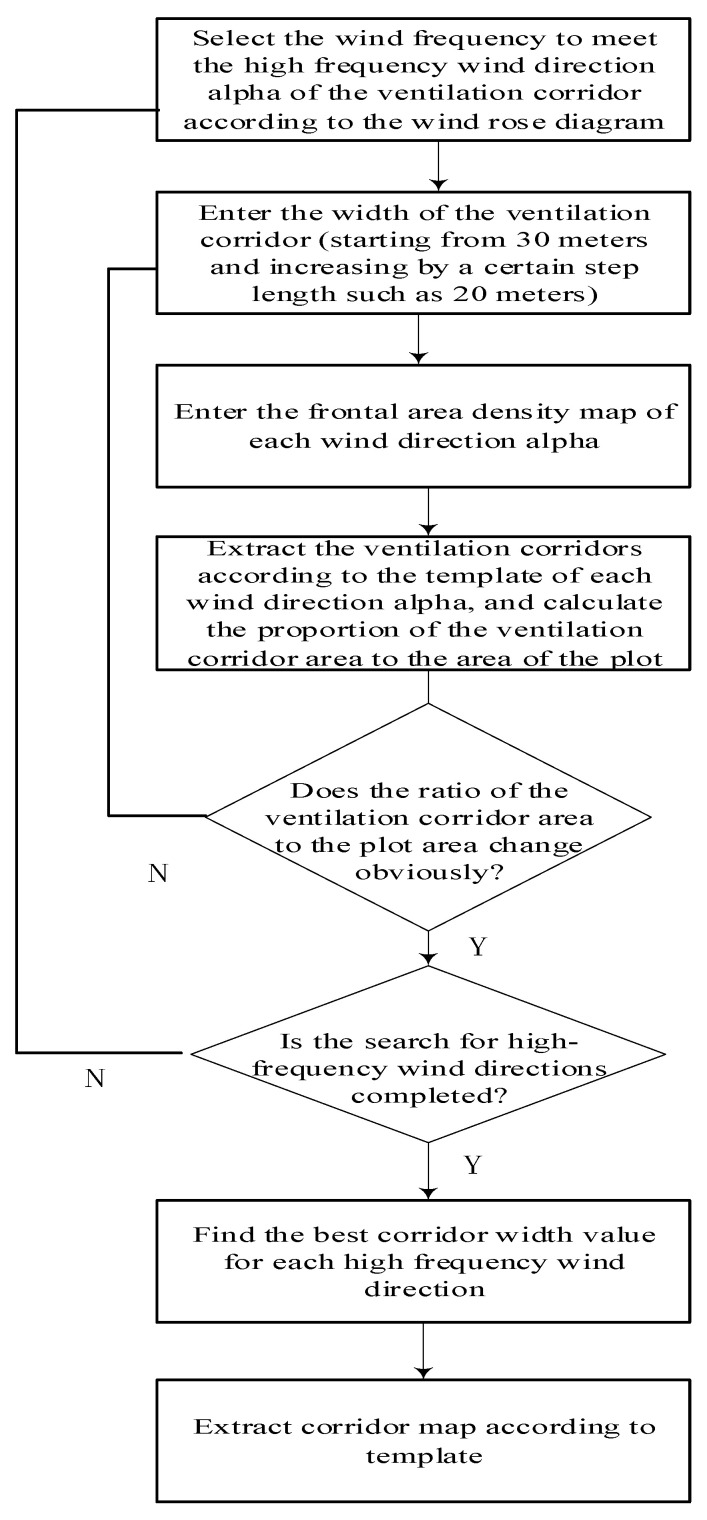
Flow chart of the optimal width for automatic mining of an urban ventilation corridor.

By using DSM–DEM map data of the tilted image, which is on the basis of the height information and wind characteristics of the city, the frontal area density of a certain dominant wind direction is calculated to form the FAD map. And by using the classification empirical value of the ventilation corridor, the template matching operation is performed to reach the ventilation corridor. The specific process is shown in Figure 12.

**Figure 13:**
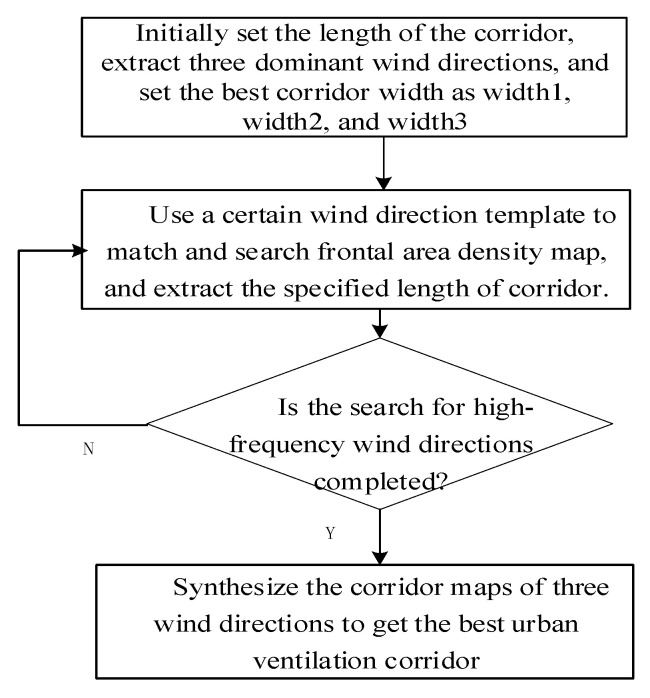
The process of extracting corridors based on the optimal corridor width.

Automatic mining process of a ventilation corridor:

(a) When excavating the corridor, the length of corridor should first be set. This paper used a conclusion from a previous study that determined the length of a corridor is greater than 500 m, and the length of a corridor generally takes 1000 m.
(b) We can choose a corridor width of one of the wind directions and use the template of the wind direction to match the frontal area density map. Then, the corridor of the alpah1 wind direction can be obtained. Finally, we can calculate the total area of the corridor (that is, the width of the corridor multiplied by the length of the corridor).
(c) We can complete the template matching of the alpha2 and alpha3 wind directions and find the best ventilation corridor, that is, the total area of the corridor reaches the maximum.
(d) Repeat step (c), complete the template matching of the alpha2 and alpha3 wind directions and determine the best ventilation corridor, that is, the total area of the corridor reaches the maximum. The specific process is shown in Figure 13.

## 5. Experiment and Result Analysis

### 5.1. Experimental Settings

In this experiment, the Quest Kodiak 100 aircraft was equipped with a five-view high-definition tilt aerial camera to obtain the tilted image data of Deqing city. After verification, the multi-view tilted image DSM map and DEM map were extracted, and the DSM–DEM map was formed into urban buildings height and ground roughness data using Global Mapper. According to the wind feature statistics of the dominant wind directions in Deqing City over the past 10 years, we projected the dominant wind direction on the DSM–DEM map in order to calculate the FAD map. Then, we performed image processing, such as binarization, expansion, corrosion, and template matching, and finally realized the automatic acquisition of urban ventilation corridor maps of various specified widths or lengths.

Experimental environment: Windows Server 2016, 64 bit operating system; internal memory of 256 GB; CPU was an Inter(R) Xeon(R) Gold 5117 CPU @2.00 GHz; MATLAB R2019; Global Mapper 17.

### 5.2. Calculation of the Maximum Effective Width of a Ventilation Corridor

In the experiment, corridors of different widths and lengths were excavated according to the dominant wind directions of the target city in winter (i.e., −67.5 degrees, −45 degrees, and −90 degrees), and the maximum effective width of the corridor was calculated.

**Figure 14:**
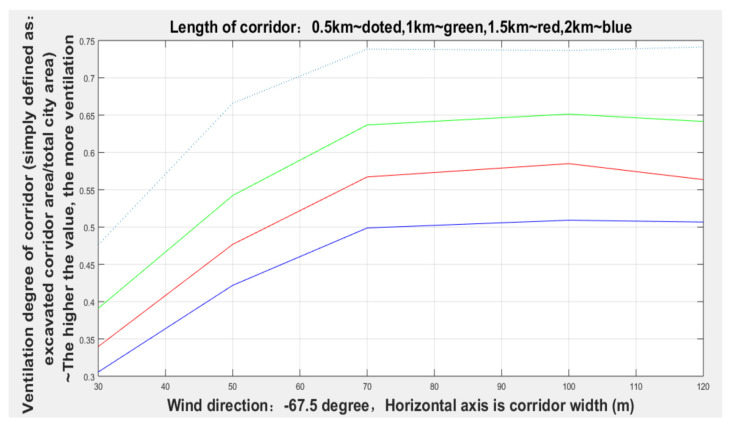
Ventilation conditions of various corridor widths with a −67.5 degree wind direction.

It can be seen from Figure 14 and Table 1 that the width of the corridor with the dominant wind direction of −67.5 degrees gradually increased from 30 to 70 m, and the degree of corridors (given lengths: 0.5, 1, 1.5, and 2 km) ventilation (excavated ventilation corridor area/total urban area) gradually increased, but when the width increased to 70 m, the degree of ventilation remained relatively stable.

**Figure 15:**
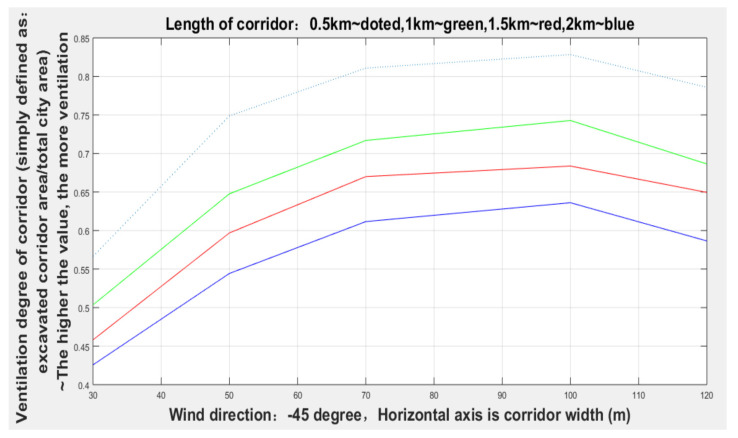
Ventilation conditions of corridors with different widths in the direction of −45 degrees.

It can be seen from Figure 15 and Table 2 that the width of the corridor with the dominant wind direction of −45 degrees gradually increased from 30 to 100 m, and the degree of corridor (given lengths: 0.5, 1, 1.5, 2, and 2.5 km) ventilation gradually increased, but when the width increased to 100 m, the degree of ventilation remained relatively stable.

**Figure 16:**
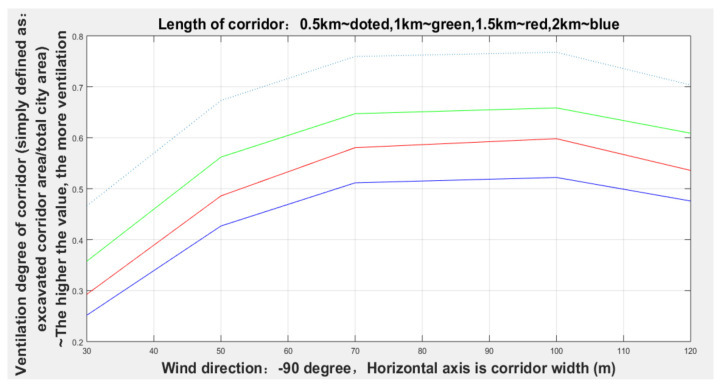
Ventilation conditions of corridors with different widths in the direction of −90 degrees.

It can be seen from Figure 16 and Table 3 that the width of the corridor with a dominant wind direction of −90 degrees gradually increased from 30 to 70 m, and the degree of corridors (given lengths: 0.5, 1, 1.5, and 2 km) ventilation gradually increased, but when the width increased to 70 m, the degree of ventilation remained relatively stable.

**Figure 17:**
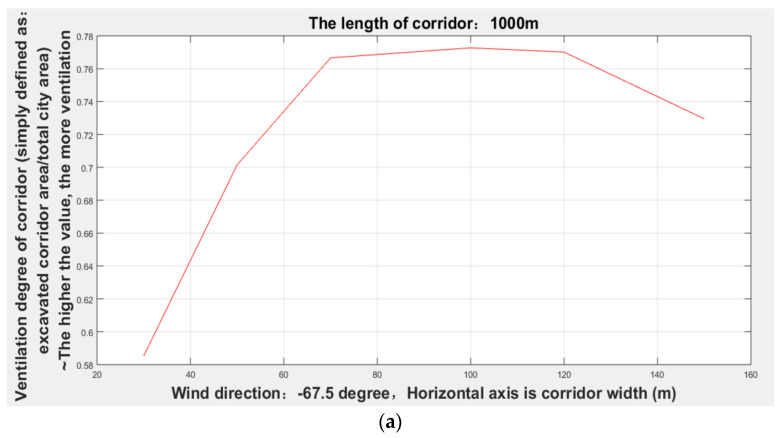

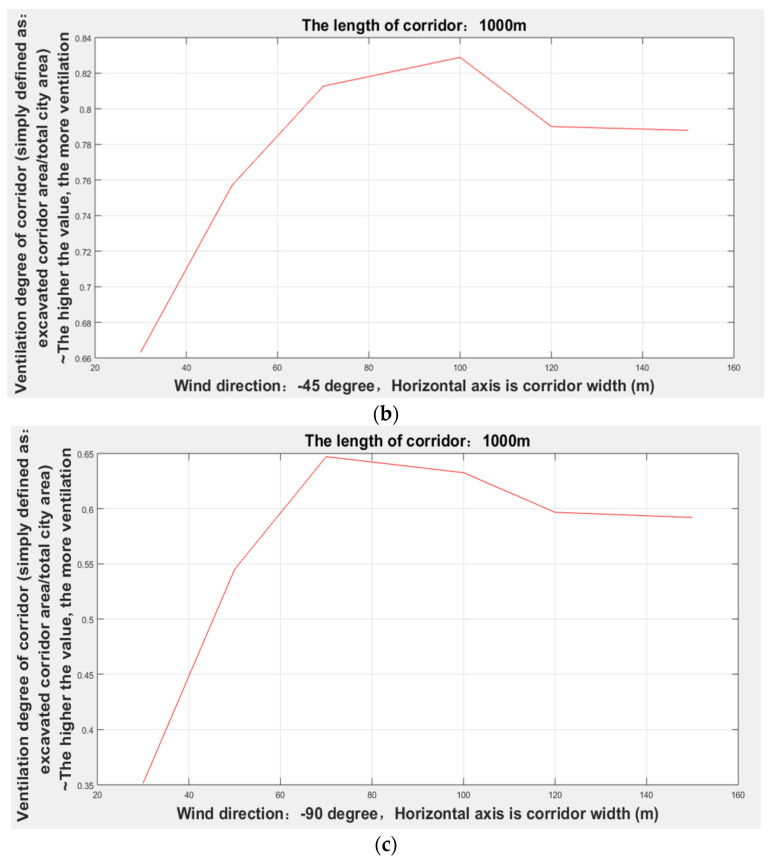
The ventilation degree of the corridor widths: (**a**) −67.5 degrees; (**b**) −45 degrees; (**c**) −90 degrees.

In order to further verify the effectiveness, that is, the existence of the maximum effective corridor width, this study tested the different widths of the 1000 m corridors with three dominant wind directions in winter as shown in Figure 17.

It can be seen from Figure 17 and Table 4 that when the wind direction is −67.5 degrees, the maximum effective corridor width is 70 m; when the wind direction is −45 degrees, the maximum effective corridor width is 100 m; when the wind direction is −90 degrees, the maximum effective corridor width is 70 m. Thus, it was verified again that there is a maximum effective ventilation corridor width.

### 5.3. Results of Ventilation Corridors

**Figure 18:**
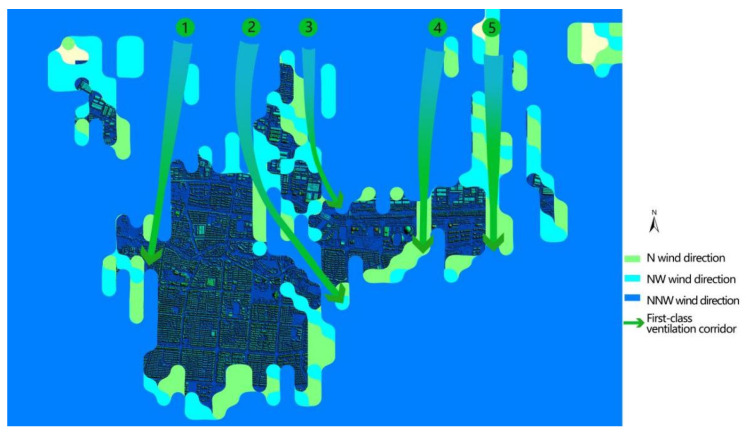
Results of the automatic mining of a 200 m wide and 500 m long corridor (digital surface model (DSM) + corridor).

We can choose the angle of the dominant wind direction in winter and take the minimum length of 500 m as the standard, then extract the ventilation corridors with widths of 200, 70, and 30 m, respectively. The results of mining with a width of 200 m (Figure 18) show that the program automatically excavated five ventilation corridors with a width of 200 m and above, which can be used as a reference for the first-level air ducts of the urban ventilation system. The northwest wind prevails in Deqing during winter. The first-level ventilation corridors follow the prevailing wind direction to introduce natural wind into urban areas. The space carriers of No. 1, No. 3, and No. 4 wind passages are railway lines and the main road of the city. The No. 2 air duct runs from the high-tech industrial development zone to the county people’s government and city parks to low-density residential land, and the No. 5 air duct enters the city along the urban road after the industrial park land was used.

The 70 m wide mining results are shown in Figure 18. Three new 70 m width ventilation corridors that introduce natural wind can be used as a reference for the secondary air ducts of the urban ventilation system. At the same time, several primary air ducts routes will be extended, and secondary air ducts are mostly urban roads relying on space carriers.

**Figure 19:**
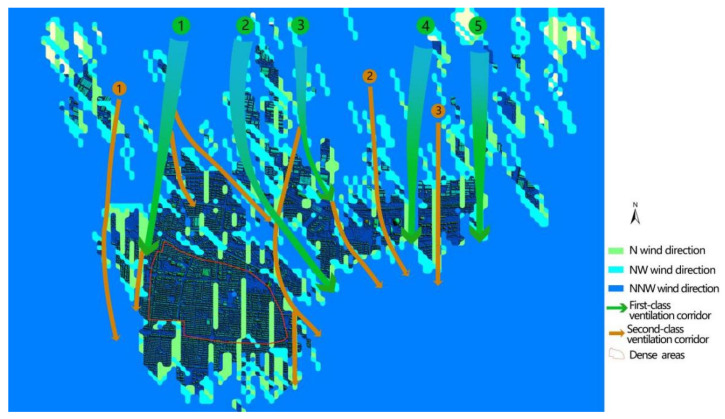
The results of the automatic mining of a 70 m width and 500 m corridor (DSM + corridor).

**Figure 20:**
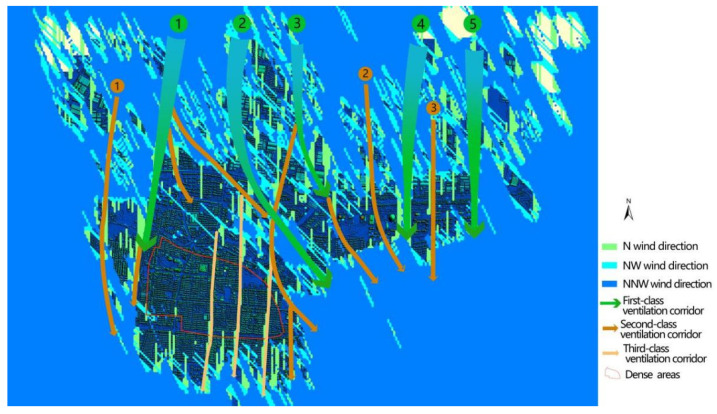
Results of the automatic mining of a 30 m width and 500 m corridor (DSM + corridor).

Based on Figure 18 and Figure 19, there are still potential tertiary wind corridors in dense areas in the southern part of the city that can be excavated at a smaller scale (30 m wide). After calculating the FAD and running the control system program, three new 30m-width corridors dependent on urban roads were excavated in these dense areas, as shown in Figure 20.

We invited three experts, who are engaged in urban planning research, to independently draw three-level corridors after analyzing the high-definition image map of Deqing city and fieldwork. After comparison, the same average ratios of the three-level corridors drawn by the experts and the corridors automatically extracted in this paper were 93.1%, 91.6%, and 90.25%, respectively.

## 6. Conclusions

Aimed at existing potential urban ventilation corridor problems, this paper extracted a multi-view oblique image DSM map and DEM map based on HD data obtained from aeromagnetic oblique photography. In addition, this paper used building elevation information, which was formed by the DSM–DEM difference map, to calculate the dominant wind direction projection to obtain a FAD map. Then, image processing techniques, such as binarization and template matching, were used to excavate urban ventilation corridors of different widths and lengths to provide technical support for scientific and reasonable planning for the city. Experiments in Deqing County, Zhejiang Province, China, proved that the method in this paper is effective. In future research, we will further utilize the roughness and other information on the urban wind environment to excavate the urban ventilation corridors more comprehensively and provide virtual reality technical support for precise microclimate environment planning and design of the city.

## Figures and Tables

**Table 1:** Ventilation degree of each corridor length and width when the wind direction was -67.5 degrees.

	Corridor Width = 30 m	Corridor Width = 50 m	Corridor Width = 70 m	Corridor Width = 100 m	Corridor Width = 120 m
Corridor length = 500 m	0.4548	0.6539	0.7212	0.7170	0.7271
Corridor length = 1000 m	0.3504	0.5282	0.6178	0.6269	0.6157
Corridor length = 1500 m	0.2879	0.4548	0.5475	0.5521	0.5290
Corridor length = 2000 m	0.2433	0.3963	0.4729	0.4689	0.4583

**Table 2:** The degree of ventilation of the length and width of each corridor when the wind direction was −45 degrees.

	Corridor Width = 30 m	Corridor Width = 50 m	Corridor Width = 70 m	Corridor Width = 100 m	Corridor Width = 120 m
Corridor length = 500 m	0.4856	0.6922	0.7610	0.7903	0.7363
Corridor length = 1000 m	0.4025	0.5681	0.6424	0.6794	0.6038
Corridor length = 1500 m	0.3322	0.5033	0.5837	0.5977	0.5532
Corridor length = 2000 m	0.2737	0.4275	0.5060	0.5231	0.4608
Corridor length = 2500 m	0.2237	0.3809	0.4618	0.4901	0.4219

**Table 3:** Ventilation degree of each corridor length and width when the wind direction was −90 degrees.

	Corridor Width = 30 m	Corridor Width = 50 m	Corridor Width = 70 m	Corridor Width = 100 m	Corridor Width = 120 m
Corridor length = 500 m	0.4584	0.6609	0.7594	0.7513	0.6959
Corridor length = 1000 m	0.3516	0.5451	0.6471	0.6326	0.5967
Corridor length = 1500 m	0.2892	0.4678	0.5805	0.5687	0.5214
Corridor length = 2000 m	0.2493	0.4110	0.5115	0.4955	0.4620

**Table 4:** Verification of the automatic mining of ventilation corridors.

	Corridor Width = 30 m	Corridor Width = 50 m	Corridor Width = 70 m	Corridor Width = 100 m	Corridor Width = 150 m
Wind direction = −67.5°	0.5853	0.7015	0.7727	0.7701	0.7295
Wind direction = −45°	0.6632	0.7569	0.8289	0.7900	0.7879
Wind direction = −90°	0.3516	0.5451	0.6326	0.5967	0.5920

## Data Availability

Not Applicable.
